# Three-dimensional chemical mapping using non-destructive SEM and photogrammetry

**DOI:** 10.1038/s41598-018-29458-8

**Published:** 2018-07-20

**Authors:** Lionel C. Gontard, Moisés Batista, Jorge Salguero, José J. Calvino

**Affiliations:** 1 Departamento de Ciencia de los Materiales e Ingeniería Metalúrgica y Química Inorgánica, Universidad de Cádiz, Puerto Real, 11510 Spain; 2 Departmento de Ingeniería Informática, Escuela Superior de Ingeniería, Universidad de Cádiz, Puerto Real, Cádiz, 11519 Spain; 3 Departamento de Ingeniería Mecánica y Diseño Industrial, Escuela Superior de Ingeniería, Universidad de Cádiz, Puerto Real, 11519 Spain

## Abstract

The *slice and view* approach in electron microscopy defines an ensemble of destructive techniques that is widely used for studying in 3D the structure and chemistry of samples with dimensions ranging from µm to mm. Here, a method is presented for measuring with high resolution and quantitatively the morphology and chemical composition of the surface of a sample in 3D. It is non-destructive and therefore, it is complementary to *slice and view* methods. The scheme is based on the fusion of conventional scanning electron microscopy (SEM) imaging, multi-view photogrammetry and compositional mapping using energy dispersive X-ray spectroscopy (EDXS). We demonstrate its potential by performing an accurate study of adhesion wear of a tungsten carbide tool that is difficult to obtain using conventional characterization techniques.

## Introduction

**Figure 1:**
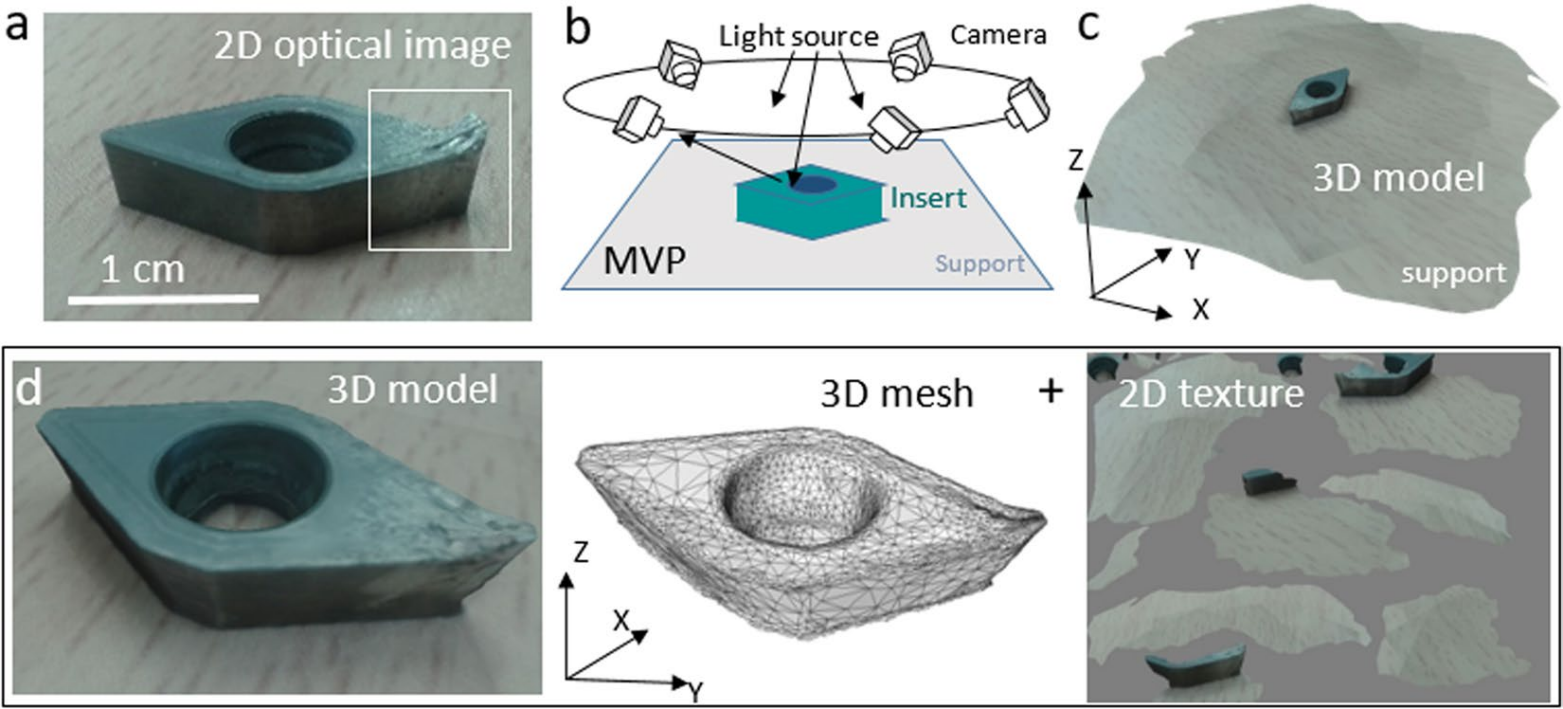
Multi-view photogrammetry of an insert tool. (**a**) Optical image of the WC-Co tool studied in this work. The white square shows wear at the tip used for machining an Al alloy. (**b**) Illustration of the geometry of acquisition of a series of images in optical photogrammetry for the three-dimensional reconstruction of the surface of the tool. (**c**) 3D model of the insert reconstructed using multi-view photogrammetry. (**d**) The 3D model of the WC-Co insert (left) is made of a mesh describing the morphology (center) that is wrapped with a texture of intensities (right) for photo-realistic appearance.

Several developments in electron microscopy and specimen preparation are enabling the 3-dimensional visualisation of specimens, and driving new advances in the structural characterization of material and biological systems. In the transmission electron microscope, electron tomography can be applied to studies of thin samples^1^. In the scanning electron microscope (SEM), serial sectioning techniques are performed *in situ* to study the inner structure of bigger samples by removing iteratively slices of material with an ion beam and imaging the cleared surface with the electron beam^2^. Photogrammetry is a non-destructive technique used with optical or SEM images to reconstruct the external shape and texture of an object albeit at the cost of not reconstructing the inner part of the sample^3–6^. In multi-view photogrammetry (MVP), collections of images acquired from multiple viewpoints can be combined using robust algorithms to extract corresponding common features in image pairs and to build a 3D point cloud gathering millions of surface points^5–7^. Then, these points are triangulated to form a 3D mesh of the surface enclosing the volume of the object that is wrapped with the texture of intensities of the original images. Figure 1 shows an example of a 3D model reconstructed using optical photogrammetry.

A fundamental difference of MVP using SEM with respect to optical methods is that the interaction of an electron beam with a target material results in a number of observable signals, such as secondary (SE), Auger (AE) and backscattered (BS) electrons, X-rays and cathodoluminescence (emission of photons from luminescent materials, CL). These SEM signals can be mapped point-by-point in two dimensions to build an image.

**Figure 2:**
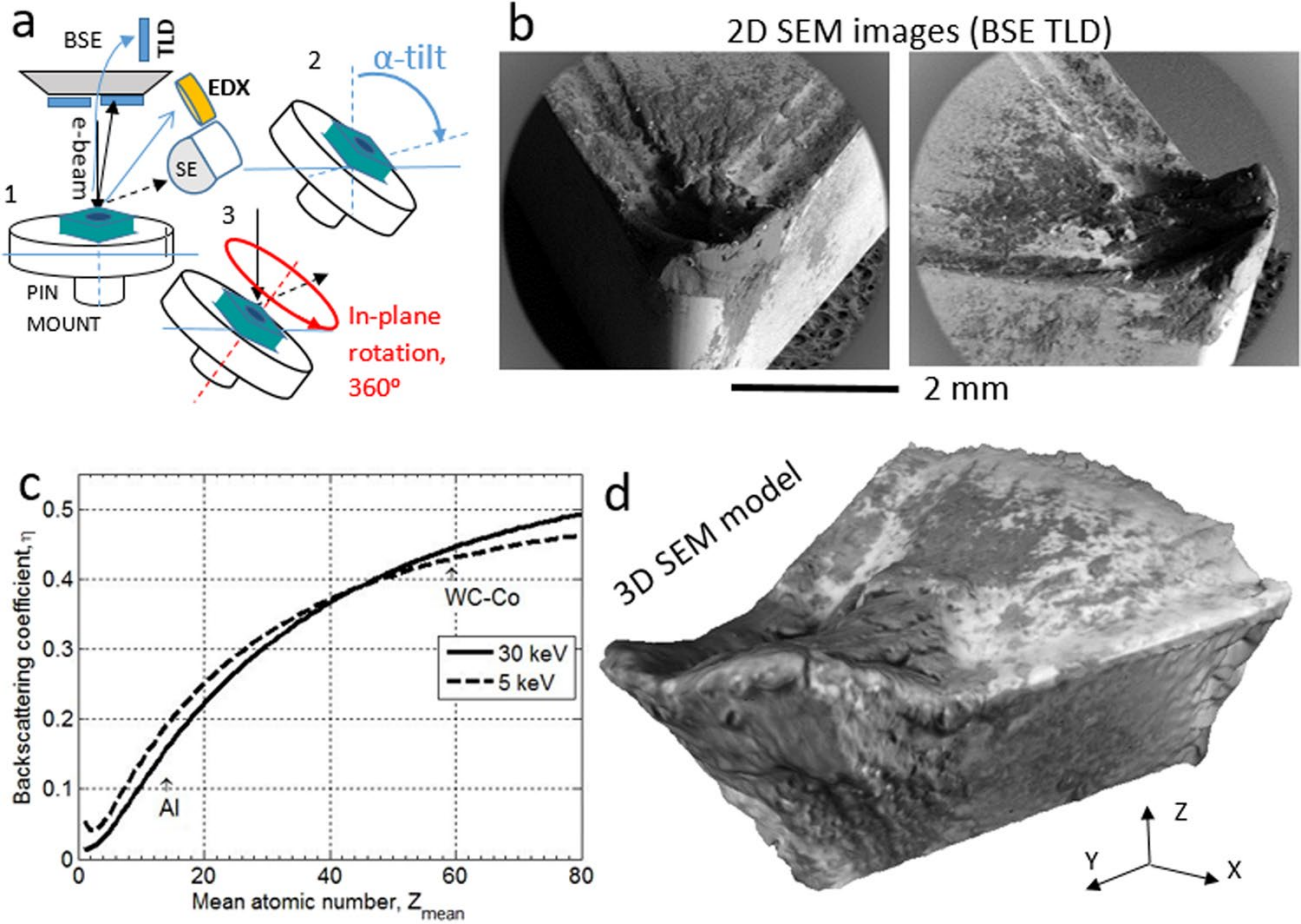
SEM/BSE photogrammetry. (**a**) In SEM, multi-view photogrammetry (MVP) can be applied too by acquiring SEM images from different viewpoints. The geometry of acquisition of the images consists in tilting and rotating in-plane the sample stage. (**b**) The 2D SEM images are representative of a series of 36 images of the cutting tool acquired with an acceleration voltage of 30 kV and using the signal of backscattered electron (BSE) measured with a through-the-lens detector (TLD). (**c**) The graph shows the dependence of the backscattering coefficient with the atomic number, for an energy of the incoming electron. When *E*_0_ is increased from 5 to 30 keV the value of *η* of the lightest element (Al) falls approximately from 0.18 to 0.14 respectively, while for WC-Co the *η*-value remains almost constant (from 0.43 to 0.44). Therefore, compositional contrast in BSE is enhanced when a higher *E*_0_ is used. (**d**) Visualization of the 3D SEM model reconstructed using MVP with a collection of BSE images as described in the main text. The darker areas (dark pixel intensities) of the texture can be interpreted qualitatively with an aluminum-rich phase. The 3D model can be explored interactively from within the section S1 of the Supplementary Material.

At first, MVP can be used for reconstructing a 3D model textured with intensities linked to chemical and optical information of the sample surface when applied to a collection of such SEM images. For example, backscattered electrons (BSEs) are those electrons from the incident electron beam that are re-emitted from the sample surface after undergoing a series of elastic scattering events that reverse their travel direction. BSE images can be acquired with high spatial resolution and large depth of field. The total number of BSEs per incident electron is called the backscattering yield or coefficient, *η*, which increases with mean atomic number Z_mean_^8–10^ (see Methods section), thus MVP can be applied to a series of images recorded with a BSE detector for reconstructing a 3D model of a sample textured with information of the surface composition (linked to Z_mean_). Unfortunately, quantitative compositional mapping from the BSE contrast alone is difficult, because of the non-linear dependence of η on the atomic number Z and the energy of the incoming electrons, *E*_0_, and the dependence of η with the surface orientation (more details can be found in Methods). In addition, different chemical phases with the same Z_mean_ number cannot be differentiated. Alternatively, quantitative chemical information can be measured in SEM as two-dimensional elemental and phase maps using energy-dispersive x-ray spectroscopy (EDXS). However, MVP reconstruction algorithms fail if a collection of EDXS maps is used as input data. The reason is that in realistic experiments (finite time) these type of maps have poor signal-to-noise ratio and little texture (see Fig. 2).

We have devised a procedure that circumvents the limitations of BSE and EDXS for reconstructing high-resolution 3D surface models textured with true quantitative chemical information using SEM and MVP. We describe the methodology, and as a proof-of-concept, we apply it for the characterization of wear of a tungsten carbide tool (WC-Co) used extensively for dry machining in the aeronautical industry. Environmental laws recommend “dry machining”, but without lubrication or a cooling agent, the subsequent increase of friction changes the tool’s surface finish and accelerates its degradation, and results in poor surface quality of the workpiece^11^. It is therefore very important monitoring the condition of the surface of these tools for optimizing their design and machining performance^12^. The study of the surface topography and chemistry of the rake face of these tools typically involve using techniques such as laser confocal microscopy^13^, white light interferometry^14^, focus-variation microscopy^15^ or laser mass spectrometry^16^. However, these techniques are limited in resolution, or provide only local information. More advanced characterization of wear in cutting tools consist in the analysis of surface changes from 2D SEM images^17^, together with the local measurement of chemical properties using 2D EDXS mapping^15,17^ and Auger electron spectroscopy (AES)^15^. We show that the multi-dimensional characterization (morphology and chemistry in 3D) of the tool using SEM and MVP provides an accurate description with high resolution of the overall geometry and chemical makeup of the sample.

## Results

Figure 1a shows a 2D optical image of the WC-Co insert examined in this work. It was used for dry roughing of a cylindrical bar made of an aluminum alloy UNS A92024-0 (Al-4% Cu). Tool wear is clearly visible at the tip used for machining (more details in Methods). A 3D model of the insert was reconstructed using optical MVP from a collection of 40 images. They were acquired from random viewpoints around the insert with the camera integrated in a mobile phone as shown schematically in Fig. 1b. The entire series of images was downloaded to a computer and a 3D model (Fig. 1c,d) was reconstructed using Agisoft Photoscan, a commercial software application for photogrammetry^18^.

The 3D model of the insert (Fig. 1c,d, left) is made of a 3D mesh (Fig. 1d, center) that describes the morphology of the surface of the tool wrapped with a 2D texture (Fig. 1d, right) built from the color intensities of the original images to achieve a photo-realistic appearance. The 3D model is limited in resolution and the intensities of the texture cannot be used for identification of the chemical phases at the surface.

Figure 2 shows the 3D model of the same WC-Co insert but this time reconstructed using a series of 36 SEM images acquired with the BSE signal in a Nova FEGSEM 450 microscope operated with an acceleration voltage of 30 kV. Figure 2a shows schematically the adapted geometry of acquisition of the images required for MVP combining tilt and rotation of the sample stage. Photogrammetry algorithms perform stitching of successive images based on their intensities; hence, they must include overlapping regions. As a rule of thumb, the same details of an area of the sample should be contained in 2 or 3 different images. A photogrammetric reconstruction often fails if the input images have little texture, and bring better results when the images have high spatial resolution, and low noise. BSE images comply with these requirements. Figure 2b shows two representative images of the insert from two viewpoints, which are optimal for successful MVP reconstruction due to their high contrast, rich in textural details and high pixel resolution. Figure 2d shows the result of the MVP reconstruction (see Methods section). In contrast with the 3D model obtained using optical MVP (Fig. 1a) the 3D model in Fig. 2d using BSE images shows high-resolution details of the morphology of the tip, and the 3D mesh of the model can be used for extracting quantitative information of different geometrical properties like in Fig. 4. More details are given in the Discussion and the Methods sections. Moreover, the gray levels of the BSEs’ intensities of the texture wrapping the 3D mesh are linked to the local density (≈Z_mean_) of the surface (BSE contrast). Under the conditions used for testing the insert tool, both the high compression forces and high temperatures softens the machined aluminum alloy, which is extruded plastically onto the tool surface. Therefore, dark patches of the texture must correspond to a higher concentration of aluminum (Z = 13, *η* = 0.14) and the brighter areas must correspond to the surface of WC-Co that is denser (Z_mean_ = 58.3, *η* = 0.44).

**Figure 3:**
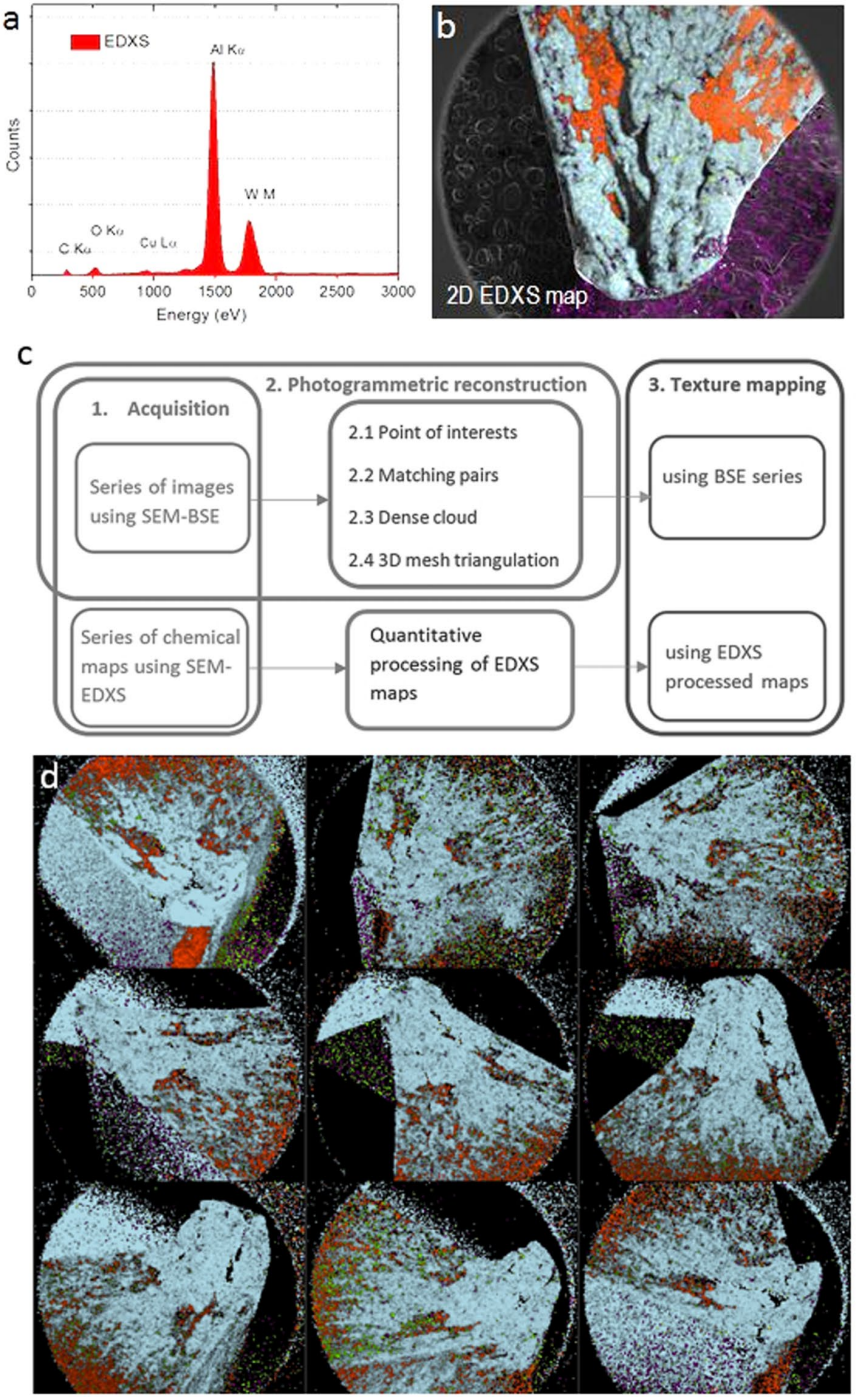
SEM/EDXS photogrammetry. (**a**) EDX spectrum of the WC-Co insert tool. (**b**) EDX map of the top surface of the insert tool overlaid on the BSE image, obtained through averaging 8 maps acquired with a dwell time of 200 µs (total time for each map ≈ 5.46 minutes). Different colors correspond to different chemical elements, W (orange), Al (light blue), C (violet). (**c**) Steps followed for performing the fusion of a 3D mesh reconstructed using BSE images with the texture reconstructed from a series of EDXS maps. (**d**) Nine representative EDXS maps from different viewpoints of a collection of 36 obtained by averaging 2 frames of 256 × 200 pixels acquired with a dwell time of 1 s (total time for each map ≈ 1.7 minutes). The series of EDXS maps was used to obtain the 3D models shown in Fig. 5.

The composition of the surface was analised also using EDXS. Figure 3a,b show respectively an EDX spectrum and an EDX map of the top surface of the WC-Co, both acquired in a Nova FEGSEM 450 equipped with a windowless x-rays detector EDAX Octane Ultra SDD. The dominant colors of each pixel in the Fig. 3b indicates the presence and distribution of W-rich (Orange color) and Al-rich (Blue color) phases. The EDXS map in Fig. 3b required a total acquisition time of 5.46 minutes. An experiment to acquire the 36 EDXS maps required for MVP would take approximately an effective acquisition time of 3.3 hours. However, such a long exposition means that a very high electron dose will be impinged to the sample and radiation damage will be more likely to occur. Even if one performs such a long experiment, EDX maps may still not be suitable for reconstruction. Photogrammetric reconstruction is based on algorithms that match the same point of the sample in different images acquired at different orientations. A chemical map like the one shown in Fig. 2b is made of pixelated areas that can artificially change between maps acquired from different viewpoints. Therefore, photogrammetric reconstruction is more reliable using BSE (or SE) images. Also, a high electron dose induces charging in samples with poor electrical conductivity. It is a phenomenon that changes the pixels intensities in a random or umpredictible way that can detriment the quality of the images and can lead to errors or failure of the photogrammetric reconstruction. In some cases the reconstruction algorithm is robust enough and cope with small amount of charging^6^, but in general if charging effects are obvious, they should minimised by coating the sample with a thin layer of conductive material, e.g, carbon or gold.

In our scheme, following the flowchart of Fig. 3c we acquired a series of EDXS maps in parallel with a BSE series of images. The series of BSE images was used for reconstructing a 3D model using MVP, and then the 3D mesh was re-textured with three series of maps obtained by processing the series of EDXS maps (full details can be found in Methods). Figure 3d, shows nine representative EDXs maps of the collection of 36 maps of the WC-Co insert acquired from different viewpoints. Figure 5 shows the 3D model of the mesh of the tool textured not with the BSE information but with three textures built from the EDXS maps, and showing the atomic percent (at%) and weight percent (wt%) distributions of several chemical elements, and the distributions of several chemical phases. We observed that the sample did not damage under intense electron irradiation, hence, the BSE images and the EDXS maps were acquired with a high probe current of 26 nA. A high current enhances the signal-to-noise and decreases the duration of the acquisition time. The acceleration voltage was set to 20 kV with the aim of increasing the spatial resolution of the EDX spectra (by decreasing the size of the interaction volume) and simultaneously having a good contrast of the BSE signal (see the curves in Fig. 2c). In general, the beam current and the acceleration voltage must be chosen in each experiment as a balance between signal-to-noise, contrast and resolution, and also radiation damage and charging of the sample.

## Discussion

The region within the white rectangle in Fig. 1b shows the deformation of the shape of the tip of the WC-Co insert, and it is due to the secondary adhesion of aluminum from the workpiece^13^. The wear model depends upon the cutting conditions used for machining the workpiece and several mechanisms have been proposed, namely abrasion (related to thermo-mechanical action), adhesion (related to micro-welding and built-up formation and removal), diffusion (chemical alteration due to atomic migration at high temperature), and fatigue. Secondary adhesion of workpiece material onto the tool, one of the mechanisms that operate in a wider range of cutting temperatures, is a cyclic dynamic process, including^19–22^: (1) the initiation of a Built-up Layer (BUL) at the tool-chip interface, (2) the growth of a Built-Up Edge (BUE) to a certain size with various irregular shapes and geometries, and (3) when BUE reaches a critical size, it plastically extends onto the tool rake face forming a new Built-up Layer (BUL)^23^, or it breaks giving rise to vibrations^24^. The BUE is extremely strain hardened and can grow up to a noticeable size and, in certain situations, it can replace the cutting tip itself to perform actual cutting. Therefore, its effect on machining performance cannot be ignored.

**Figure 4:**
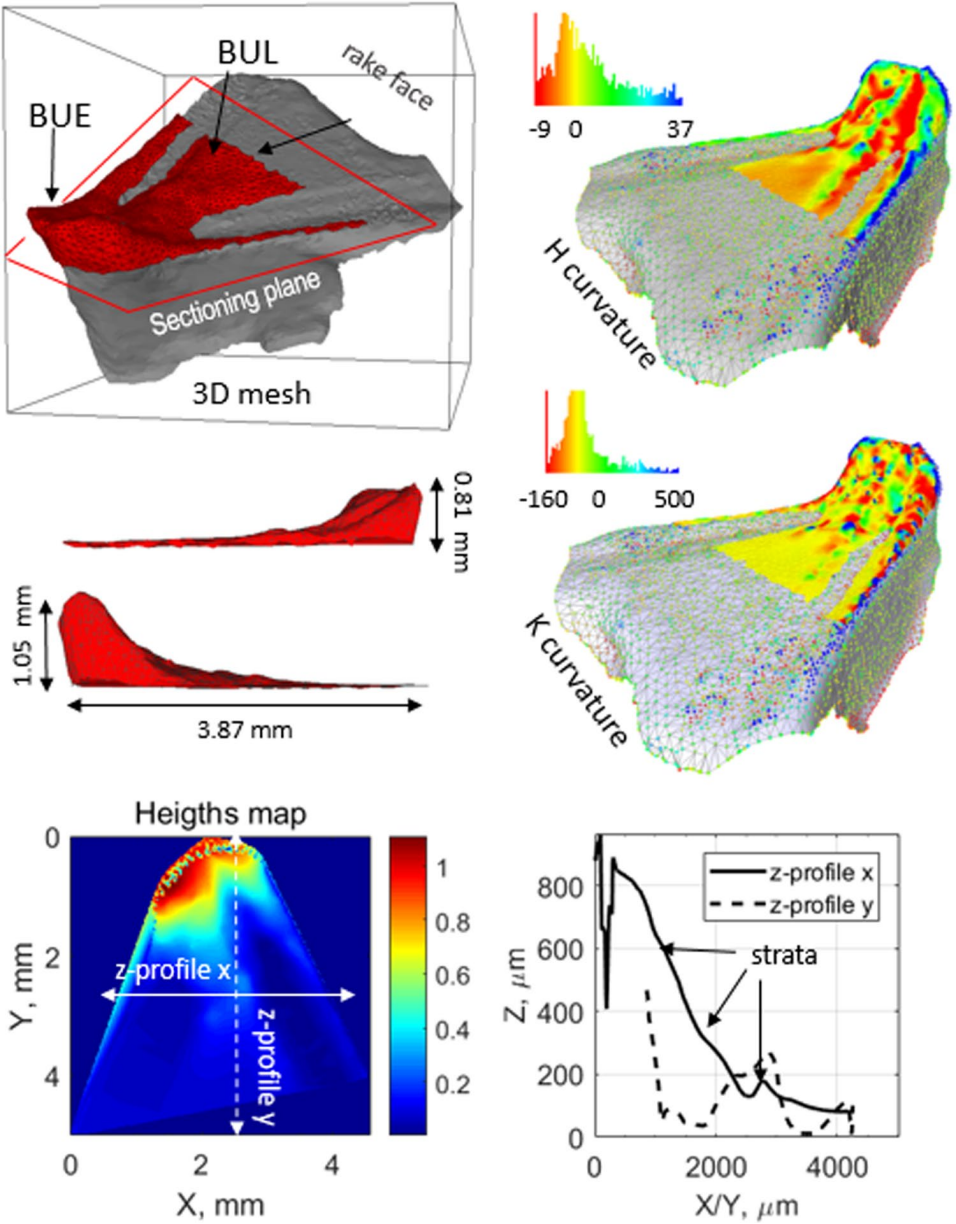
Morphological characterization. Mesh of the 3D model of the tool tip. The aluminum-rich BUE/BUL formed by adhesion of aluminum can be segmented from the 3D mesh (in red color). As explained in the main text and in the Methods section, different geometrical measurements can be performed on the segmented model: dimensions, heights map, rugosity parameters, local curvature (Gaussian and Mean curvatures), and the topological genus.

The 3D mesh of the WC-Co insert can be used to measure the geometry of the worn tip (Fig. 4). For example, the BUE/BUL of aluminum can be segmented via software by sectioning the 3D mesh with a plane parallel to the top surface or rake face of the tool and several types of measurements can be done, such as the volume (5.8 10^8^ μm^3^) and the surface (11.6 mm^2^) of the adhered material. Using the sectioning plane as the reference of zero level, the segmented mesh can be converted to a heights map from which depth profiles and roughness parameters (R_a_ = 73.7 µm, R_q_ = 12 µm and R_sk_ = 0.084 µm) can be measured (see Methods). The BUE that forms at the edge of the tool by mechanical adhesion reaches a maximum height of 1.05 mm. Far from the edge on the rake face, the BUL that is formed by plastic extrusion reaches a maximum distance of 3.87 mm while its thickness decreases forming stratified layers that are linked to the cyclic process of formation of secondary adhesion^15^. Local geometry of the BUE/BUL can be assessed also from the 3D mesh as shown in Fig. 4. For example, the Gaussian curvature K = *κ*_1_·*κ*_2_ and mean curvature H = ½ (*κ*_1_ + *κ*_2_) can be determined, where the principal curvatures *κ*_1_ and *κ*_2_ measure the maximum and minimum bending of a regular surface at each point. A positive K implies that the surface is locally a peak or a valley whereas a negative K indicates a saddle point. If K ≠ 0, its magnitude gives some information about ‘how far from developable’ the surface is, i.e., how much stretching will be needed to form it. If the stretching is too much, the material will tear. Likewise, curvatures can provide insight into the operating thermodynamics that operates in the geometry of a microstructure through H and K^25^. The 3D mesh supplies also information about global topology, a field of geometry of increasing interest in materials science^26,27^. Topology studies invariance of certain properties under smooth continuous mechanical deformation and it is characterised by an integer parameter called genus, *g*. The genus is related to the Euler characteristic *χ* of a surface *χ* = 2 (1-*g*), which in turn can be calculated like *χ* = V − E + F, the number of (V)ertices, (E)dges and (F)aces of the 3D mesh respectively. For the BUE/BUL in Fig. 4, χ = 2 (V: 1911, E: 5727, F: 3818) and *g* = 0.

**Figure 5:**
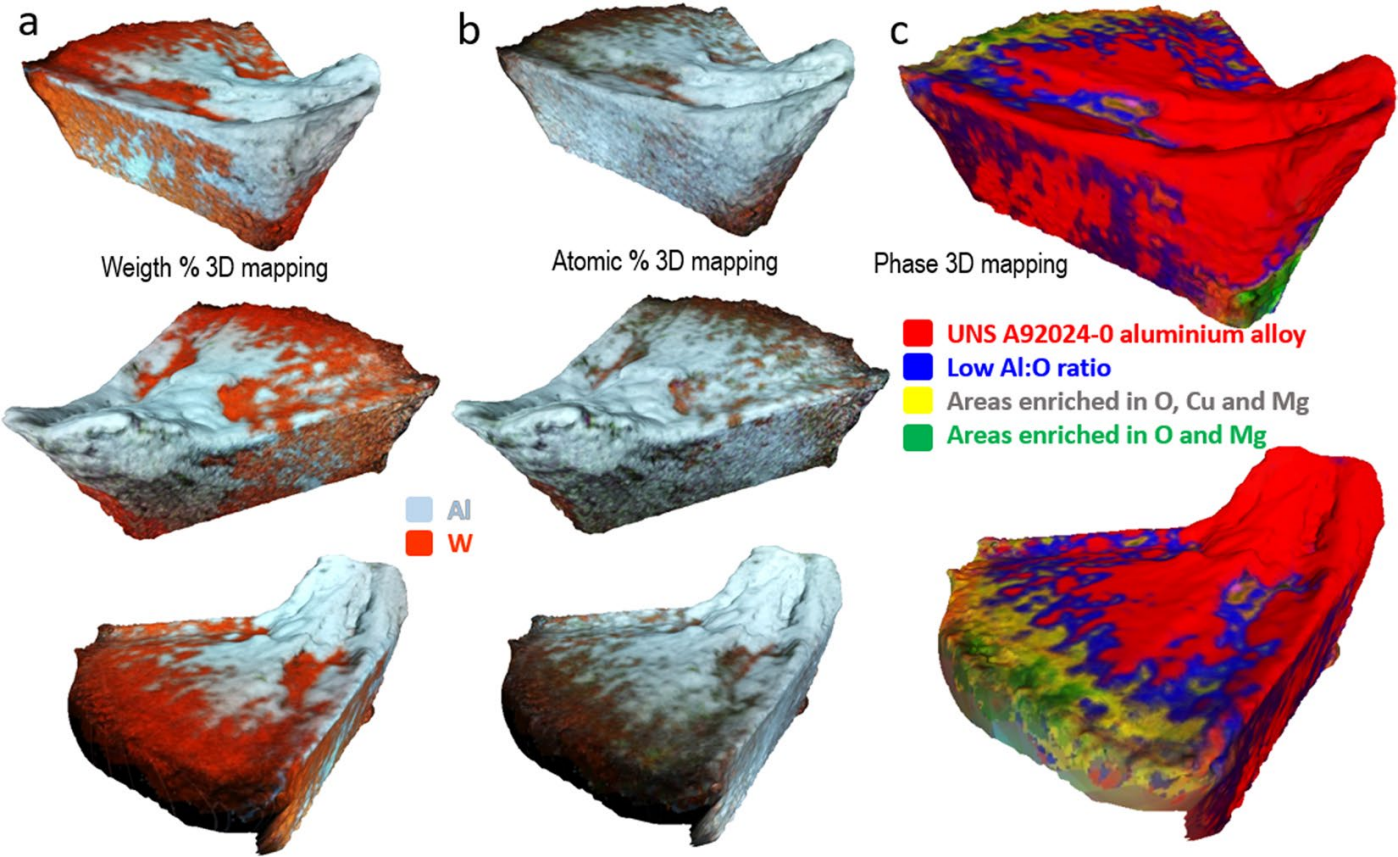
Multi-dimensional mapping. High-resolution 3D models and chemical information of the surface of a WC-Co insert after dry machining a UNS A92024-0 Al alloy. A series of 36 SEM images using a BSE detector and a series of 36 EDXS maps were acquired concurrently at 20 kV from the same viewpoints of the cutting tool. The 3D mesh (Fig. 4) of the insert was reconstructed from the series of BSE images. Finally, the 3D mesh was textured with three series of images obtained by processing semi-quantitatively the EDXS maps. The figure shows the visualization from three viewpoints of the 3D mesh textured with Atomic %, Weight % and phases. The resulting 3D models can be explored interactively from within the section S1 of the Supplementary Material. (**a**) The model clearly shows the distribution of the secondary adhesion wear. (**b**) A thin layer of Al covers large areas of the insert. (**c**) The content ratios of O, Mg and Cu and Al are different at the edge, the flank and the primary and secondary rake face of the tool.

Metrology on 3D models requires an evaluation of errors. Photogrammetric algorithms automatically calculate the viewpoints from which the images were acquired in a process called bundle adjustment. When the images do not overlap sufficiently or are textureless, or noisy, errors on the calculation of the correct coordinates of the viewpoints occurs. It is one of the most important sources of error, and can distort heavily the shape of the model affecting the reliability of the technique or it can lead also to reconstruction failure. We have compared the reliability of the 3D mesh obtained with MVP with a 3D model of the WC-Co insert measured using x-ray Computed Tomography (CT). CT scan is a non-destructive characterisation technique that is frequently used to study samples in 3D with dimensions that can range from mm until meters. The CT scan of the tool is included in the Supplementary Material S2, and consists of a tomogram that contains full 3D volume information, not only surface information. The 3D models using MVP and SEM in Figs 2–5 are clearly superior in terms of resolution and contains much less artefacts. We can say that MVP is more reliable than CT for studying surfaces of samples with sizes under the milimeter.

Furthermore, the textures of the 3D models (Figs 2 and 5) provide an overview of the elemental composition and phases of the surface of the tool linked to the local geometry. That information is not accessible from x-ray CT or from single EDXS maps. The 2D maps in Fig. 3d contain areas with zero counts due to shadowing of the x-ray detector by the sample. Note also, that the textures in Fig. 5 are made by blending (averaging) out several EDX acquired from different perspectives of the sample. Thus, the 3D models contain information that is more complete and accurate when compared with the information of a single 2D map.

Finite element simulations using the cutting conditions of the dry turning test show that the tip of the WC-Co tool heat up to temperatures between 40–300 °C (see details in Methods) that in turn, softens the alloy and facilitates the flow by extrusion of the aluminum alloy over large distances on the surface of the tool. The history of the sample treatment can be used for interpreting the contrast of the texture, for instance the dark patches visible on the texture built using the BSE images (Fig. 2) must be the extruded aluminum.

On the other hand, a 3D model of the tip textured with spectroscopic information contains much more information. The wt% elemental maps like the one in Fig. 5a shows conclusively and precisely the composition of the surface and confirms that the dark patches are aluminum rich phases. The high temperatures reached during machining may favor also diffusion of aluminum over large distances. This phenomenon is supported by the 3D model textured with the at% information in Fig. 5b. It shows that aluminum, which has a high degree of chemical affinity towards cemented carbide, covers most of the surface of the tool. When the temperature of the cutting process decreases the metal become solid and is open to oxidation. The 3D phase map model in Fig. 5c shows that at the BUE the content ratio Al:O is high and decreases towards the BUL as the thickness of the adhesion layer diminishes. This thickness-chemical information provided by the 3D model can be interpreted as the surface oxidation of the aluminum. Also, the 3D phase map shows that the built-up layer on the rake face of the tool has two distinct compositions of sticking work material. At the BUE close to the tip of the cutting tool the composition is similar to the original A92024-0 alloy, with a 3.8% of Cu, 1.5% of Mg and traces (<0.5%) of other elements. In contrast, the composition Al-Cu-Mg of the thinnest regions of the BUL correspond to phases with high concentrations of Mg and Cu (up to 10% and 20% respectively). These Mg- and Cu-rich phases have been observed on alloys subjected to a tempering process^13^. The UNS A92024-0 alloy contains an α phase that corresponds to the solid solution of Cu and other alloying elements in the FCC (face centered cubic) lattice of aluminum. When these alloys are heated secondary phase particles (inclusions) can grow, in particular, Al_2_CuMg (S-phase) and CuAl_2_^28,29^. During machining the visco-plastic state of the aluminum allows welding of the alloy on the tool surface. Metallic chips of the workpiece can drag off larger intermetallic particles (which have a much higher melting point) from the BUE and redeposit them onto the rake face. When the temperature decreases the sticking of alloying elements with the aluminum and the bonding of alloying elements to the surface tool is favored.

## Conclusions

Characterising interface boundary conditions between tool and workpiece in machining is critical to the determination of the temperatures and stresses required to understand cutting tool wear and to design improved tools^13,14,17^. The multi-dimensional surface characterization demonstrated here as a proof-of-concept provides a description of the overall geometry and chemical makeup of secondary adhesion wear of a WC-Co insert that is not accessible with the use of conventional 2D elemental maps or images. In addition, 3D meshes of the inserts may be used as input models to investigate tribological properties of the tools (temperature, pressure, friction) under different cutting conditions using finite element modelling^30^. This type of analysis at different stages of machining can certainly help to a better understanding of the dynamic BUL/BUE formation and of the tool-chip contact phenomena.

The non-destructive SEM methodology presented in this work for 3D characterisation (morphology and chemical mapping) of the surface of materials can be applied potentially to many other types of material samples, at very different dimensional scales with high resolution. With little investment, it provides a novel metrological technique to the toolbox of materials scientists based on a widely available instrumentation. Further development of the technique may include the addition of markers (for example, fibbed onto the sample or onto the support if a FIB SEM is available) to facilitate the camera (viewpoint) alignment during the bundle adjustment of the images. We also stress that the method can be readily applied with no changes for 3D mapping of other types of spectroscopic signals available in SEM such as those measured using cathodoluminescence and Auger electrons detectors.

## Methods

### Material and machining conditions

3D measurements were performed on an uncoated tungsten carbide (WC-Co) interchangeable insert tool SECO® with ISO reference DCMT 11T308-F2-HX. An image of the insert is shown in Fig. 1a. WC-Co is actually a ceramic/metal composite, where metallic cobalt acts as a binding (matrix) material to hold the WC particles in place. The tool was used for dry roughing of a cylindrical bar made of an aluminum alloy UNS A92024-0 (Al-4% Cu). Four cylindrical turning tests of 120 mm were achieved on a horizontal lathe, EmcoTurn 242 TC model, equipped with a Numerical Control Emcotronic TM02 with a 2(1/2) axis. Machining parameters: cutting speed of 190 m/min, a feed of 0.4 mm/rev and a cutting depth of 1 mm.

### Reconstruction of 3D models using SEM, BSE and multi-view photogrammetry

The WC-Co tool was fixed to a pin mount using carbon tape, the sample stage of the Nova FEGSEM was tilted to 50°, and 36 BSE images of 1024 × 800 pixels were recorded rotating the sample in plane in steps of 10° between consecutive images. Figure 2b shows two representative SEM images of the insert acquired from two different viewpoints with the BSE detector and the acceleration voltage set to 30 kV for enhancing compositional contrast. Finally, the 3D model of the insert shown in Fig. 2d was reconstructed using Agisoft PhotoScan^18^ using the following parameters: (1) the images were aligned using a key point limit of 20, a tie point limit of 4, and an adaptive camera model fitting. (2) The dense cloud of points was built using a moderate depth filtering. (3) The 3D mesh was built from the dense cloud with a face count of 6.000 and interpolation. And (4), texture mapping was done using a blending mode of averaging and a UV map size of 1024 × 1024 pixels.

Alternatively, one can use open source software like the Python Photogrammetry Toolbox^31^. Note that for MVP the values of the parameters set are not as important as acquiring a good series of overlapping BSE images with texture, contrast, low noise, and minimum amount of charging.

### Reconstruction of 3D models with chemical information from EDXS

The results shown in Fig. 5 were obtained using the following steps (shown schematically in the Fig. 3c):

i. The insert was fixed onto an SEM pin mount using carbon tape. To fit the insert in the field-of-view a magnification of 19X was used.
ii. The pin mount in the SEM was inclined to an α-tilt angle of 52°.
iii. 36 BSE images of 1024 × 800 pixels and 36 EDXS maps of 256 × 200 pixels were recorded concurrently by rotating in-plane pin mount in steps of 10°, with a Nova FEGSEM 450 at 20 kV and using high current (spot 6, size of 8.2 nm and a current of 26 nA). A lower acceleration voltage reduces the interaction volume providing better resolution. The BSE signal was collected with a through-the-lens detector (TLD) located inside the gun-lens system with the suction tube set to collect backscattered electrons (BSEs). In these conditions, EDXS maps summed over two frames with a dwell time of 1 ms were acquired at each orientation of the sample. The acquisition of each map took 1.7 minutes.
iv. The 36 BSE images were binned by two (averaging) using ImageJ^32^ to a final size of 512 × 400 pixels. The series of resized images and saved with.png format was loaded into the commercial software Agisoft Photoscan and used to reconstruct the 3D mesh of the cutting tool using the same parameters used for reconstructing the model shown in the Fig. 2.
v. Each EDXS map was processed offline using the software TEAM^33^ from EDAX. Semi-quantitative analysis was done in order to generate maps of the Net intensities, Atomic % and Weight % using a pixel binning of 2 and considering only the chemical elements present in larger quantity: W, C, Co, O, Al, Cu and Mg. We also mapped the chemical phases present at the surface as combinations of Al, Cu and Mg using a feature of TEAM software called Smart Mapping and with a binning of 4 pixels. This tool uses spectra at each pixel as the basis for characterizing the elemental associations and distribution by tracking variations in component peak intensity to provide correlations and discrimination of the data between phases. Each phase map was exported as an image with.png format. The three series of maps must be resized to match the dimensions of the BSE images used for reconstructing the 3D mesh. Hence, the images were resized by a factor of 2 with bilinear interpolation to a final size of 512 × 400 pixels using ImageJ.
vi. As a result of the processing of the EDXS maps for each orientation of the sample, we obtained three series of 36 images. Finally, each of these series containing all the chemical information were used for generating three textures of the 3D mesh of the carbide tool previously reconstructed using the BSE detector. The 3D models can be explored interactively from within the section S1 of the Supplementary Material.

### Roughness measurements

For the visualization and segmentation of the 3D models we used the freeware Meshlab^34^. The segmented model of the BUE/BUL was exported into a file with.xyz format using the freeware Meshlab. This format is a plain text file with three columns corresponding to the *x*, *y* and *z* coordinates of each of the corners of the triangles that made up the triangular mesh of vertex and faces of the 3D model of the surface. The .xyz file was then read and processed with Matlab^35^ software. The point cloud (*x*_*i*_, *y*_*i*_, *z*_*i*_) of points of the mesh was rotated in 3D space until the cutting plane was parallel to the *xy* coordinates plane. Next, from the point cloud the continuous surface was recalculated using the Matlab function *TriScatteredInterp (x*, *y*, *z)*. The surface was then reprojected in the *z*-direction to obtain an elevation map or 2D heights map^7^. Finally, the parameters for rugosity were calculated using the following definitions, with *n* the number of points of the heights map and *z*_*i*_ the height at point *i*.

- Roughness average: $${{\rm{R}}}_{a}=\frac{1}{n}{\sum }_{{\rm{i}}=1}^{{\rm{n}}}|{z}_{i}|$$
- Root mean square (RMS) roughness: ${{\rm{R}}}_{q}=\sqrt{\frac{1}{n}{\sum }_{{\rm{i}}=1}^{{\rm{n}}}{{z}_{i}}^{2}}$
- Skewness: $${{\rm{R}}}_{sk}=\frac{1}{{{{\rm{R}}}_{q}}^{3}}[\frac{1}{n}{\sum }_{{\rm{i}}=1}^{{\rm{n}}}{{z}_{i}}^{3}]$$

### Backscattering electron yield

The curves in Fig. 2c were calculated as follows. BSE yield or coefficient *η* is not constant with incident electron energy, *E*_0_, but varies in a manner that depends on both the energy and *Z*, the atomic number^8^. Typically BSEs will undergo several inelastic collisions during their time inside the solid sample, and so will typically have an energy that is lower than *E*_0_, upon their escape. The energy spread of BSEs is peaked typically somewhere between 0.4 *E*_0_, and 0.95 *E*_0_ so that varies depending on the atomic number of the sample material. Analytical expressions that predict the values of *η* as a function of both *Z*, and *E*_0_ (in kilo-electron volts) have been derived^8,9^:

$$\eta (Z,\,{E}_{0})=\frac{{I}_{bks}}{{I}_{0}}={{E}_{0}}^{m(Z)}\,C(Z)$$

being

$$m(z)=0.1382-\frac{0.9211}{{Z}^{0.5}}$$

and

$$C(z)=0.1904-0.2236\,\mathrm{ln}\,Z+0.1292{(\mathrm{ln}Z)}^{2}+0.01491{(\mathrm{ln}Z)}^{3}$$

For a general sample, when the sample is not a pure element, *Z* is the mean value of atomic number for the compound $Z={\sum }_{i=1}^{n}{c}_{i}{Z}_{i}$ being *c*_*i*_ the mass concentration coefficients and ${\sum }_{i=1}^{n}{c}_{i}=1$.

### Finite element modelling

We performed FEM in order to estimate the maximum temperatures reached on the insert. The simulations were carried out with the software DEFORM-3D^36^ from Scientific Forming Technologies Corporation (SFTC) using a minimum mesh size of 0.012 mm for the UNS A92024 and 0.05 mm for the uncoated insert. The cutting conditions used were a cutting speed of 190 m/min, a feed of 0.4 mm/rev and a cutting depth of 1 mm, for a total distance of 25 mm.

### Data Availability Statement

- The datasets generated during and/or analysed during the current study are available from the corresponding author on reasonable request.
- The main results are included in this published article (and its Supplementary Information files).

## Electronic supplementary material

Dataset S1 and S2
