# Supplementary material for: Three-dimensional chemical mapping using non-destructive SEM and photogrammetry
Source: Sci Rep. 2018 Jul 20;8:11000. doi: 10.1038/s41598-018-29458-8 (PMC6054630; doi:10.1038/s41598-018-29458-8)

Supplementary material

Three-dimensional chemical mapping using non-destructive SEM and photogrammetry

Lionel C Gontard [a,b,], M Batista [c], J Salguero [c], JJ Calvino [a]*

S1. Interactive 3D models of the insert

1. 3D model textured with BSE signal (Figure 2d)
2. 3D model textured with Weight % (Figure 5a)
3. 3D model textured with Atomic % (Figure 5b)
4. 3D model textured with Phases (Figure 5c)

NOTE: The models can be rotated interactively within the pdf file clicking and moving the mouse

3D Weight %

3D Atomic %

3D Phase Map

S2. Computed tomography scan of the insert

We have compared the data obtained using 3D SEM with a reconstruction of the WC-Co using x-ray computed tomography (CT). CT is a non-destructive 3D technique widely used for inspection and metrology in several industries.

A 3D model of the insert was reconstucted using a CT scan GE x-cube Compact 195 from General Electric operated at 62 kV and with a current of 0.47 mA. The measurements were carried out at the Centre of Advanced Manufacturing for Aeronautics (CFAA) at the University of the Basque Country (Spain). The figure below shows a visualization of the 3D mesh of the surface of the WC-Co insert obtained by extracting an isosurface of the tomogram reconstructed using CT with the software VGSTUDIO from Volume Graphics. The resolution of the mesh is low, and most of the details of the BUE/BUL are not resolved.

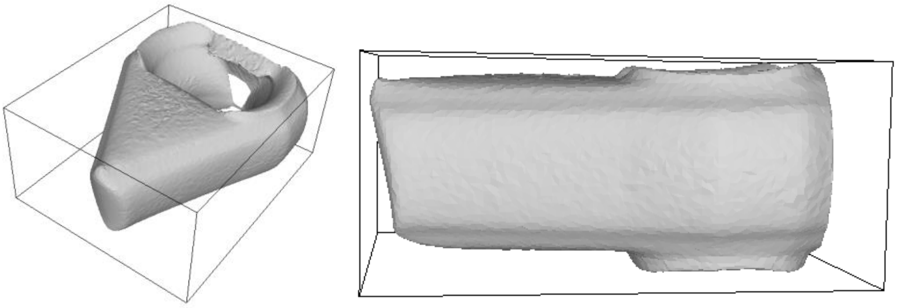

The figure below shows two orthogonal sections of the tomogram. It displays artefacts like ill-defined edges with bright intensities that are not real and blurring. CT that is widely used for the characterization of larger pieces, provides much poorer spatial resolution than 3D SEM for the characterization of the surface of smaller objects. And it does not provide chemical information.

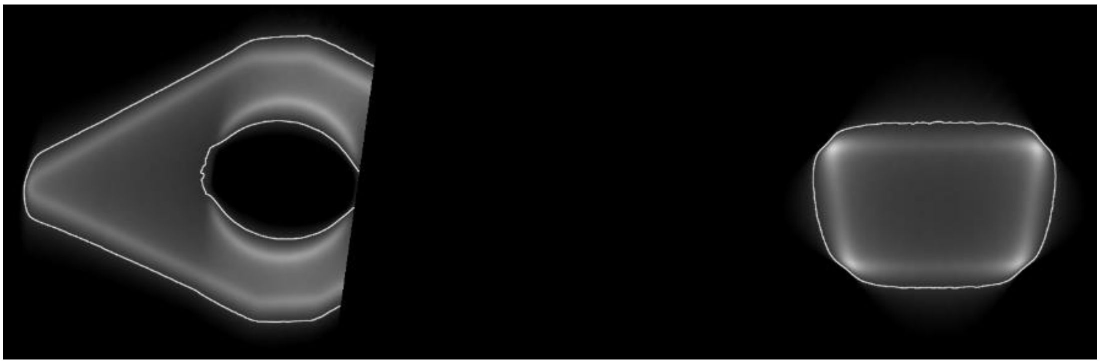

Supplement: Supplementary file 1 — Dataset S1 and S2 [file 41598_2018_29458_MOESM1_ESM.pdf]
